# Prognostic value of heart rate variability in patients with coronary artery disease in the current treatment era

**DOI:** 10.1371/journal.pone.0254107

**Published:** 2021-07-02

**Authors:** Antti O. Vuoti, Mikko P. Tulppo, Olavi H. Ukkola, M. Juhani Junttila, Heikki V. Huikuri, Antti M. Kiviniemi, Juha S. Perkiömäki

**Affiliations:** 1 Research Unit of Internal Medicine, Medical Research Center Oulu, University of Oulu and Oulu University Hospital, Oulu, Finland; 2 Biocenter Oulu, University of Oulu, Oulu, Finland; International University of Health and Welfare, School of Medicine, JAPAN

## Abstract

Coronary artery disease (CAD) mortality has declined substantially over the past decades thanks to advancing medical and interventional/surgical treatments; therefore, the prognostic value of the heart rate variability in CAD in the current treatment era is not well established. We evaluated the prognostic significance of baseline heart rate variability in 1,757 ARTEMIS study patients with angiographically verified CAD. During an average follow-up time of 8.7 ± 2.2 years, a total of 285 (16.2%) patients died. Of the patients, 63 (3.6%) suffered sudden cardiac death or were resuscitated from sudden cardiac arrest (SCD/SCA), 60 (3.4%) experienced non-sudden cardiac death (NSCD), and death attributable to non-cardiac causes (NCD) occurred in 162 (9.2%) patients. For every 10 ms decrease in standard deviation of normal to normal intervals the risk for SCD/SCA, NSCD and NCD increased significantly: HR 1.153 (95% CI 1.075–1.236, p<0.001), HR 1.187 (95% CI 1.102–1.278, p<0.001) and HR 1.080 (95% CI 1.037–1.125, p<0.001), respectively. The natural logarithm of the low-frequency component of the power spectrum and the short-term scaling exponent of the detrended fluctuation analysis also had significant association with all modes of death (p<0.001). After relevant adjustment, standard deviation of normal-to-normal intervals retained its association with NSCD and NCD (p<0.01), the natural logarithm of the low-frequency component of the power spectrum with all modes of death (p from <0.05 to <0.01), and the short-term scaling exponent of the detrended fluctuation analysis with SCD/SCA (p<0.05) and NCD (p<0.001). In conclusion, impairment of many measures of heart rate variability predicts mortality but is not associated with any specific mode of death in patients with stable CAD during the current treatment era, limiting the clinical applicability of heart rate variability to targeting therapy.

## Introduction

Cardiovascular diseases still remain the leading cause of death in western societies and coronary heart disease (CHD) is the most common cause of death, accounting for approximately 40% of cardiovascular disease deaths [1,2]. Moreover, 80% of sudden cardiac deaths (SCD) occur in CHD patients and these patients are therefore at high risk of a sudden life-threating event [3]. Coronary artery disease (CAD) patients’ autonomic nervous system abnormalities can be evaluated non-invasively using different domains of heart rate variability (HRV).

The standard deviation of normal-to-normal intervals (SDNN) is a classic time-domain measurement of HRV. It reflects total variation of the heart rate signal, and reduced values of SDNN may indicate increased sympathetic activity or decreased parasympathetic activity [4] and have been associated with cardiac and other adverse outcomes [5,6]. High-frequency component of HRV power-spectrum (HF) reflects parasympathetic while low-frequency component (LF) reflects parasympathetic and sympathetic autonomic nervous control [7]. There are plenty of studies that have demonstrated the prognostic value of the frequency-domain measurements of HRV in different populations; for example, in one study in postinfarction patients, there was significant difference in LF component of HRV between those who died and survivors, whereas the difference in HF component was less marked [8]. The LF/HF ratio, a measurement of sympathovagal balance [9] and very low-frequency (VLF) component of HRV power spectrum have also been shown to yield prognostic information in postinfarction patients [10]. Some previous studies have suggested that some of the nonlinear HRV measurements may be slightly better predictors of mortality in postinfarction patients than the traditional measurements [11–13]. The short-term scaling exponent of detrended fluctuation analysis (DFA1) describes the short-term fractal-like scaling properties of the RR interval time-series and is caused by complex interaction between the parasympathetic and sympathetic nervous system. Simultaneous sympathetic and vagal stimulation decreases DFA1 [14]. Reduced DFA1 has been shown to be associated with the risk of ventricular arrhythmias and death in various study populations [13,15]. The slope of the relationship between spectral power and frequency on bi-logarithmic scale shows a linear portion between 10^−4^ and 10^−2^ Hz (PL slope). The PL slope reflects the long-term scaling characteristics of HRV in the region of ultralow- and very-low-frequency bands [16]. A steep PL slope has been observed to be a better predictor of all-cause mortality or arrhythmic death than the conventional power spectral bands in postinfarction patients [11]. The data on the prognostic significance of approximate entropy (ApEn), a complexity measure of HRV, are limited. Decreased ApEn of HRV has been shown to precede spontaneous episodes of atrial fibrillation in patients without structural heart disease [17] and in patients after coronary artery bypass surgery [18].

In the modern treatment era, CHD mortality has declined substantially, and it has been estimated that around 45% of the decline is due to medical and interventional/surgical treatments [19,20]. However, the effect of this decline of mortality on the prognostic value of HRV in CAD patients is not clear. Furthermore, it is not well established whether decreased HRV is a general indicator of mortality risk or whether it is more closely associated with the risk of SCD. Therefore, the aim of the present study was to evaluate the value of conventional and nonlinear measurements of HRV in predicting mortality in patients with angiographically verified CAD in the modern treatment era.

## Methods

### Study population

The ARTEMIS study is a prospective observational study (Innovation to Reduce Cardiovascular Complication of Diabetes at the Intersection NCT01426685) that recruited patients between August 2007 and December 2012 at the Division of Cardiology in the University Hospital of Oulu [21,22]. The study included 1,946 patients with angiographically confirmed CAD defined as at least 50% stenosis in one or more major coronary vessels. Risk assessments were done 3 to 6 months after angiography. Of the study subjects, 833 had type 2 diabetes mellitus (DM), which was defined according to the WHO guidelines [23]. At the initial examination visit, laboratory analyses were done after overnight fasting using standard measures, and medical therapy for CAD and DM was optimized. Exclusion criteria were New York Heart Association class IV or Canadian Cardiovascular Society (CCS) class IV, planned or existing implantable cardioverter-defibrillator (ICD), permanent pacemaker, age under 18 or over 80 years, life expectancy <1 year due to any comorbidities, pregnancy, end-stage renal dysfunction requiring dialysis, or being otherwise unfit for the study due to physical or psychological condition. The study patients were contacted by mailed questionnaires and telephone calls to inquire about possible interim hospitalization at least at 2 and 5 years of follow-up. The final adjudication of the cause for hospitalization was based on diagnoses from medical records [21]. Due to CAD, patients were also under regular control by their doctors. Of the 1,946 patients, 189 did not undergo 24-hour ambulatory ECG or were excluded from the analysis as a result of technical and biological disturbances. Therefore, the total number of patients in the present study was 1,757, of whom 729 had DM. All subjects included in the study gave informed consent. The study was approved by the institutional Ethics Committee of Northern Ostrobothnia Hospital District and complied with the Declaration of Helsinki.

### Endpoints of the present study

The endpoints of the present study were sudden cardiac death or resuscitated cardiac arrest (SCD/SCA), non-sudden cardiac death (NSCD), and non-cardiac death (NCD). The SCD/SCA group included patients who suffered SCD or were resuscitated from sudden cardiac arrest, whichever occurred first, and the criteria for SCD were witnessed death within 1 hour from the onset of symptoms or death occurring within 24 hours of last witnessed moment of being alive. NSCD included the patients who experienced death for cardiac reason but did not fulfill the criteria of SCD/SCA. The NCD group included all patients who suffered death due to other than cardiac reasons. The endpoints were determined from emergency rescue reports, hospital and physician reports, autopsy data, death certificates, and interviews with next of kin. The cause and mode of death were adjudicated by two independent investigators, and if needed, disagreement or uncertainty was resolved in consultation with the investigators (M.J.J. and H.V.H.) [21]. Autopsy data were available in most cases of SCD because according to the law, medicolegal autopsy is mandatory in Finland in cases of unexpected death.

### Electrocardiography and heart rate variability

A digital Holter recorder (Medilog AR12; Huntleigh Healthcare Cardiff, U.K.) with an accuracy of 250 Hz was used to perform 24-hour ambulatory ECG recordings. During recording, patients were advised to carry on with their normal daily activities without limitations. The recordings were processed and analysis was conducted using HEARTS software (Heart Signal Oy, Oulu, Finland). Inter-beat intervals (R-R intervals) were edited visually using the interpolation method to remove all artefacts and ectopic beats from data. In this method, removed R-R intervals are replaced with a local average of the previous accepted normal R-R intervals [24]. In the present study, the following HRV parameters were included in the analysis as they represent widely different conventional and nonlinear domains of HRV and are most frequently used in clinical studies. Of the conventional HRV domains, SDNN was used as a time-domain measure and the included frequency-domain measures were the natural logarithm (ln) of HF power (ln(HF)) (0.15–0.4 Hz), ln of LF power (ln(LF)) (0.04–0.15 Hz), and ln of VLF power (ln(VLF)) (0.0033–0.04 Hz) of power spectrum and LF/HF ratio [9]. Nonlinear methods included DFA1, PL slope and ApEn. The detrended fluctuation analysis quantifies the presence or absence of the fractal-like correlation properties in RR-interval time series. In this method, root-mean-square is calculated from RR-interval time series which are integrated, divided to specific window sizes, and detrended. After repeating the steps mentioned above using different window sizes, HRV is plotted on a log-log scale as a function of the window size. DFA1 is the slope of this line in the window sizes ≤11 RR-intervals [25]. The PL slope method assesses the long-term fractal-like properties of the HRV [11]. ApEn is used to assess the complexity or regularity of the heart rate dynamics [26].

### Echocardiography

Echocardiographic examinations were conducted using the General Vivid 7 ultrasound instrument (General Electric Healthcare, Little Chalfont, UK) to evaluate left ventricular ejection fraction (LVEF) and left ventricular mass index (LVMI). LVEF was determined using two-dimensional mode and LVMI was obtained by calculating left ventricle mass using the recommended formula and dividing it by body surface area [27].

### Statistical analysis

Continuous variables are presented as mean ± standard deviation and categorical variables as percentages. The statistical significance of differences between baseline clinical characteristics was assessed using the standard two-tailed t-test for continuous variables and Chi-square test or Fisher’s exact test for dichotomous variables, as appropriate. The baseline clinical characteristics that differed significantly in univariate comparison were entered into the multivariate Cox regression analysis and the most predictive clinical model was defined individually for each endpoint group. HRV measurements that differed significantly in the baseline comparison were tested in the multivariate Cox regression analysis in the defined clinical model one at a time as continuous variables. Patient-specific follow-up times were individually applied for time-specific analyses due to varying follow-up times. A *p*-value < .05 was considered statistically significant. Association of HRV measurements with clinical risk indicators was assessed using the Pearson correlation coefficient between continuous variables and the two-tailed t-test between continuous and dichotomous variables. The receiver operating characteristics curves were used to optimize the cut-off points for HRV measurements. Kaplan-Meier curves were formed to show the cumulative proportional probabilities for different modes of death and Log rank test was used to assess the statistical significance of the separation of the curves. Analyses were conducted using IBM SPSS version 25 (IBM, Armonk, NY, USA).

## Results

During a follow-up period of 8.7 ± 2.2 years, a total of 285 (16.2%) patients died in the study group of 1,757 patients. Of the patients, 63 (3.6%) suffered SCD/SCA and 60 (3.4%) NSCD. Death attributable to non-cardiac reasons occurred in 162 (9.2%) patients.

### Baseline clinical characteristics of study patients

Many of the baseline clinical characteristics differed significantly between the patients who experienced a certain mode of death compared with the patients who remained alive/experienced a different mode of death (Table 1). The patients who suffered SCD/SCA were slightly older, had more commonly DM, were more often smokers, had more commonly CCS class ≥2, larger left ventricular end-systolic diameter, lower LVEF and higher LVMI compared with patients who remained alive/experienced other mode of death. Patients who died due to NSCD were older, had more commonly DM, consumed less alcohol, had more frequently CCS class ≥2, larger left ventricular end-systolic diameter and higher LVMI in comparison with subjects who remained alive/experienced other mode of death. Patients who experienced NCD were older, were more often males, had more commonly DM, were less often smokers, consumed less alcohol, had more commonly CCS class ≥2, had more often right bundle branch block and smaller left ventricular end-diastolic diameter compared with patients who remained alive/experienced other mode of death.

**Table 1:** Clinical characteristics and heart rate variability parameters in study patients at baseline.

Variable	Alive* (n = 1472)	SCD/SCA (n = 63)	*p*	NSCD (n = 60)	*p*	NCD (n = 162)	*p*
Age	65.5 ± 8.4	68.9 ± 7.8	0.024	73.0 ± 7.6	<0.001	72.0 ± 7.3	<0.001
Gender M, (%)	66.3	76.2	0.170	66.7	0.889	76.5	0.013
DM, (%)	38.6	65.1	<0.001	65.0	<0.001	50.0	0.024
BMI (kg/m^2^)	28.3 ± 4.4	28.8 ± 4.8	0.327	29.5 ± 5.7	0.100	27.6 ± 4.2	0.053
BP, systolic	147.1 ± 24.1	147.6 ± 24.7	0.961	148.2 ± 24.2	0.803	150.6 ± 28.6	0.141
BP, diastolic	80.6 ± 11.2	80.9 ± 10.9	0.831	78.9 ± 11.1	0.249	81.3 ± 13.5	0.508
Smoker (%)	8.5	17.5	0.032	5.0	0.630	6.2	0.020
Alcohol	2.3 ± 4.8	3.3 ± 7.5	0.357	0.7 ± 1.6	<0.001	1.6 ± 3.7	0.045
CCS ≥ 2 (%)	38.0	65.1	<0.001	73.3	<0.001	50.6	0.012
LBBB (%)	2.6	4.8	0.244	3.5	0.667	2.5	1.000
RBBB (%)	4.1	6.3	0.528	3.5	1.000	8.3	0.025
PCI/CABG (%)	80.3	88.9	0.104	81.7	0.870	77.8	0.405
β-blocker (%)	87.2	93.7	0.173	93.3	0.230	89.5	0.532
Ca-blocker (%)	23.5	28.6	0.453	33.3	0.093	25.3	0.773
ACEI/ARB (%)	67.5	77.8	0.099	75.0	0.262	65.4	0.480
Lipid l.m. (%)	92.7	85.7	0.097	85.0	0.088	88.3	0.099
Anti-t.m. (%)	97.8	98.4	1.000	93.3	0.041	99.4	0.255
Anti-a.m. (%)	0.7	0.0	1.000	3.3	0.080	0.6	1.000
Diuretics (%)	28.6	41.3	0.129	56.7	<0.001	48.8	<0.001
Nitrates (%)	33.1	54.0	0.002	61.7	<0.001	40.7	0.168
LVEDD (mm)	50.3 ± 5.8	52.2 ± 9.2	0.101	52.0 ± 8.2	0.103	48.9 ± 6.6	0.002
LVESD (mm)	32.0 ± 5.9	35.7 ± 9.9	0.005	35.4 ± 9.5	0.008	31.2 ± 6.9	0.063
LVEF (%)	64.7 ± 8.4	59.8 ± 12.6	0.004	61.3 ± 13.0	0.057	65.1 ± 9.7	0.305
LVMI (g/m^2^)	105.8 ± 26.1	119.8 ± 32.8	0.003	120.0 ± 34.1	0.004	109.8 ± 26.7	0.231
HR	66 ± 8	68 ± 11	0.073	63 ± 9	0.024	66 ± 9	0.587
SDNN (ms)	139.9 ± 41.2	117.6 ± 38.5	< 0.001	114.0 ± 41.9	<0.001	126.6 ± 41.6	<0.001
ln(HF)	5.41 ± 0.95	5.18 ± 1.23	0.079	5.32 ± 1.26	0.631	5.38 ± 1.18	0.804
ln(LF)	5.98 ± 0.84	5.44 ± 1.12	0.001	5.40 ± 1.06	<0.001	5.59 ± 1.00	<0.001
LF/HF ratio	2.21 ± 1.56	1.76 ± 1.39	0.063	1.35 ± 1.06	<0.001	1.56 ± 1.14	<0.001
ln(VLF)	6.94 ± 0.66	6.50 ± 0.86	<0.001	6.40 ± 0.84	<0.001	6.61 ± 0.74	<0.001
DFA1	1.16 ± 0.21	1.02 ± 0.24	<0.001	1.01 ± 0.24	<0.001	1.04 ± 0.24	<0.001
PL slope	-1.34 ± 0.18	-1.41 ± 0.18	0.008	-1.44 ± 0.17	<0.001	-1.39 ± 0.18	0.001
ApEn	0.90 ± 0.20	0.93 ± 0.23	0.294	0.91 ± 0.23	0.748	0.90 ± 0.24	0.983

The values are mean ± SD or percentages. Abbreviations: ACEI/ARB = angiotensin-converting enzyme inhibitor or angiotensin receptor blocker, Alcohol = alcohol consumption in restaurant portions/week, Anti-a.m. = anti-arrhythmic medication, Anti-t.m. = anti-thrombotic medication, ApEn = approximate entropy, BMI = body mass index, BP = blood pressure, CCS = Canadian Cardiovascular Society grading of angina pectoris, DFA1 = the short-term fractal scaling exponent of detrended fluctuation analysis, DM = diabetes mellitus type 2, Gender M = male gender, HR = heart rate, LBBB = left bundle branch block, Lipid l.m. = lipid lowering medication, ln(HF) = natural logarithm of high-frequency power, ln(LF) = natural logarithm of low-frequency power, ln(VLF) = natural logarithm of very-low-frequency power, LVEDD = left ventricular end-diastolic diameter, LVEF = left ventricular ejection fraction, LVESD = left ventricular end-systolic diameter, LVMI = left ventricular mass index, NCD = non-cardiac death, Nitrates = long-acting nitrates, NSCD = non-sudden cardiac death, PCI/CABG = prior percutaneous coronary intervention or coronary artery bypass graft, PL slope = Power-law slope, RBBB = right bundle branch block, SCD/SCA = sudden cardiac death or sudden cardiac arrest, SDNN = standard deviation of normal to normal intervals, Smoker = current smoker.

*In the statistical analysis, the patients with each mode of death were compared with patients who remained alive/experienced other mode of death.

### Association of baseline heart rate variability measurements with occurrence of different modes of death

The values of SDNN, ln(LF), ln(VLF), DFA1 and PL slope were lower in patients with each mode of death when compared with other patients. The patients who suffered NSCD or NCD had lower values of LF/HF ratio when compared with patients who remained alive/experienced other mode of death. The values of ln(HF) or ApEn did not differ significantly between patients who experienced any mode of death and other patients (Table 1).

### Clinical models for different modes of death after adjustments

Clinical variables that differed significantly in univariate comparison (Table 1) were entered into the Cox multivariate regression analysis to define the most predictive clinical model individually for each endpoint group (Table 2). When age, DM, smoking, CCS class ≥2, left ventricular end-systolic diameter, LVEF and LVMI were tested in the Cox multivariate clinical model in a stepwise manner, age, DM, smoking, CCS class ≥2, LVEF and LVMI retained a significant association with SCD/SCA after adjustments. When age, DM, alcohol consumption, CCS class ≥2, left ventricular end-systolic diameter and LVMI were entered to the Cox multivariate clinical model in a stepwise manner, age, DM, CCS class ≥2 and left ventricular end-systolic diameter retained a significant association with NSCD. When age, gender, DM, smoking, alcohol consumption, CCS class ≥2, right bundle branch block, left ventricular end-diastolic diameter were tested in the Cox multivariate clinical model in a stepwise manner, age, gender, DM and left ventricular end-diastolic diameter remained significant predictors of NCD.

**Table 2:** Univariate and multivariate predictors of sudden cardiac death or sudden cardiac arrest, non-sudden cardiac death and non-cardiac death.

		SCD/SCA			NSCD			NCD		
Variable		HR	95% CI	*p*	HR	95% CI	*p*	HR	95% CI	*p*
Age	uv	1.041	1.009–1.075	0.012	1.131	1.088–1.175	<0.001	1.104	1.080–1.129	<0.001
	mv	1.040	1.004–1.078	0.031	1.125	1.082–1.170	<0.001	1.110	1.085–1.135	<0.001
Gender M	uv	1.607	0.900–2.869	0.109	1.074	0.622–1.855	0.797	1.658	1.153–2.384	0.006
	mv							2.436	1.675–3.543	<0.001
DM	uv	2.689	1.601–4.516	<0.001	2.534	1.484–4.329	<0.001	1.437	1.056–1.956	0.021
	mv	2.294	1.357–3.879	0.002	2.314	1.347–3.977	0.002	1.510	1.107–2.059	0.009
Smoker	uv	2.551	1.260–5.165	0.009	0.612	0.186–2.010	0.419	0.916	0.471–1.782	0.796
	mv	2.449	1.112–5.395	0.026						
Alcohol	uv	1.034	0.987–1.083	0.161	0.849	0.736–0.979	0.024	0.962	0.918.1.009	0.113
	mv									
CCS ≥ 2	uv	2.942	1.751–4.943	<0.001	4.433	2.494–7.880	<0.001	1.684	1.237–2.294	<0.001
	mv	2.037	1.183–3.505	0.010	2.612	1.447–4.717	0.001			
RBBB	uv	1.595	0.579–4.392	0.367	0.915	0.223–3.755	0.902	2.206	1.250–3.893	0.006
	mv									
LVEDD	uv	1.052	1.012–1.093	0.010	1.050	1.009–1.093	0.016	0.963	0.938–0.989	0.005
	mv							0.969	0.943–0.995	0.019
LVESD	uv	1.074	1.042–1.108	<0.001	1.069	1.035–1.104	<0.001	0.974	0.948–1.000	0.054
	mv				1.070	1.039–1.102	<0.001			
LVEF	uv	0.948	0.927–0.970	<0.001	0.958	0.935–0.982	<0.001	1.001	0.983–1.020	0.879
	mv	0.968	0.945–0.992	0.008						
LVMI	uv	1.016	1.008–1.023	<0.001	1.016	1.009–1.024	<0.001	1.006	1.001–1.012	0.023
	mv	1.009	1.000–1.018	0.049						
SDNN	uv	1.153	1.075–1.236	<0.001	1.187	1.102–1.278	<0.001	1.080	1.037–1.125	<0.001
	mv	1.064	0.993–1.141	0.079	1.105	1.027–1.189	0.007	1.066	1.020–1.113	0.004
ln(LF)	uv	1.864	1.428–2.433	<0.001	1.937	1.472–2.549	<0.001	1.576	1.329–1.868	<0.001
	mv	1.324	1.015–1.725	0.038	1.427	1.101–1.850	0.007	1.349	1.134–1.604	<0.001
LF/HF ratio	uv	1.242	1.005–1.536	0.045	1.914	1.410–2.598	<0.001	1.472	1.262–1.717	<0.001
	mv	1.098	0.901–1.338	0.353	1.284	0.975–1.691	0.075	1.289	1.107–1.501	0.001
ln(VLF)	uv	2.123	1.556–2.898	<0.001	2.453	1.808–3.329	<0.001	1.776	1.450–2.175	<0.001
	mv	1.327	0.960–1.834	0.086	1.586	1.152–2.182	0.005	1.597	1.277–1.996	<0.001
DFA1	uv	1.255	1.137–1.385	<0.001	1.294	1.171–1.431	<0.001	1.229	1.154–1.308	<0.001
	mv	1.122	1.009–1.247	0.034	1.092	0.976–1.222	0.125	1.143	1.070–1.221	<0.001
PL slope	uv	1.228	1.063–1.418	0.005	1.344	1.161–1.555	<0.001	1.163	1.063–1.273	<0.001
	mv	1.092	0.947–1.260	0.226	1.143	0.992–1.317	0.064	1.090	0.990–1.200	0.078

Abbreviations: CI = confidence interval, HR = hazards ratio, mv = multivariate, uv = univariate. Other abbreviations are the same as in Table 1. SDNN (every 10 decrease), LF/HF (every 1 decrease), ln(LF) (every 1 decrease), ln(VLF) (every 1 decrease), DFA1 (every 0.1 decrease), PL slope (every 0.1 decrease). *p*-values when compared with all other study patients, i.e., with patients who remained alive/experienced other mode of death. Only the mv HRs that remained significant after relevant adjustments are shown for clinical variables whereas all the mv HRs are shown for heart rate variability parameters. Significant clinical variables in the multivariate clinical model were age, DM, smoking, CCS class ≥2, LVEF and LVMI for SCD/SCA; age, DM, CCS class ≥2 and LVESD for NSCD; and age, gender, DM and LVEDD for NCD.

### Heart rate variability measurements in predicting different modes of death in the Cox regression analysis

**Fig 1:**
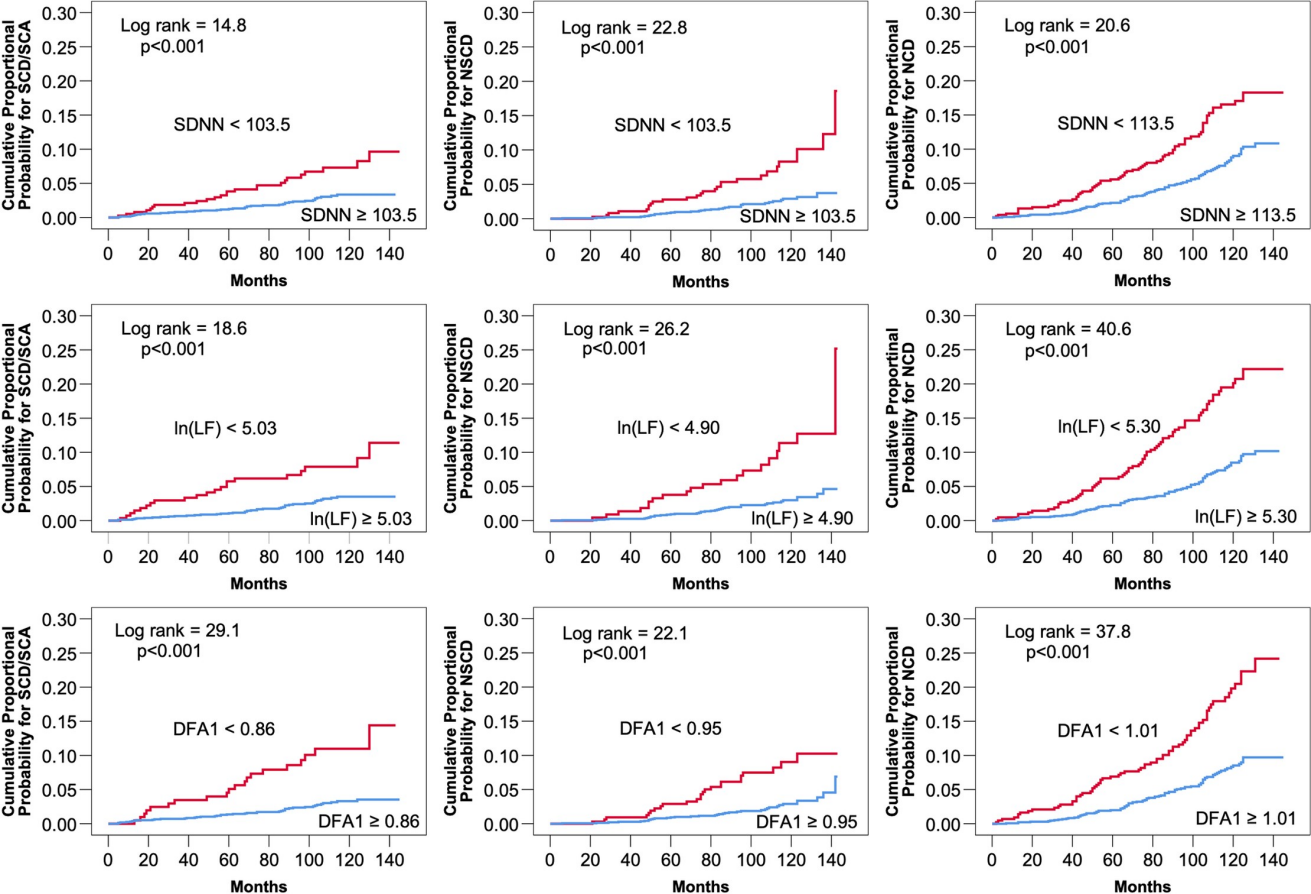
Cumulative proportional probabilities for sudden cardiac death or sudden cardiac arrest (SCD/SCA), non-sudden cardiac death (NSCD), and non-cardiac death (NCD).

HRV measurements that differed significantly between patients with any mode of death and the other patients in univariate comparison were tested one at a time in the clinical multivariate Cox hazard model (Table 2). Lower values of ln(LF) and DFA1 retained significant association with SCD/SCA after adjustments. However, ln(LF) was also associated with all other modes of death and DFA1 with NCD after adjustments. After relevant multivariate adjustments, SDNN and ln(VLF) remained associated with NSCD and NCD and LF/HF ratio with NCD. The PL slope lost its significant association with the occurrence of different modes of death after relevant adjustment in the clinical multivariate model. Cumulative proportional probabilities of SCD, NSCD and NCD in relation to SDNN, ln(LF) and DFA1 are shown in Fig 1. The cut-off points were obtained from receiver operating characteristics curves.

The cut-off points for the standard deviation of RR intervals (SDNN), the natural logarithm of low-frequency power of heart rate variability power spectrum (ln(LF)), and the short-term scaling exponent of the detrended fluctuation analysis (DFA1) were optimized from receiver operating characteristics curves. The log rank test was used to assess the statistical significance of the separations of the curves.

### Association of heart rate variability measurements with clinical risk indicators

Associations of heart rate variability measurements with clinical risk indicators are shown in Table 3. Lower values of SDNN, ln(LF) and DFA1 were associated with higher age and lower LVEF, and lower values of DFA1 with larger LVMI. Females and patients with DM or CCS class ≥ 2 had lower values of SDNN, ln(LF) and DFA1, and smokers had lower values of SDNN.

**Table 3:** Association of heart rate variability measurements with clinical characteristics.

	SDNN		ln(LF)		DFA1	
**Variable**	**r**	***p***	**r**	***p***	**r**	***p***
Age	-.085	<0.001	-.221	<0.001	-.312	<0.001
LVESD	-.044	0.066	-.035	0.146	-.033	0.165
LVEF	.087	<0.001	.095	<0.001	.051	0.035
LVMI	.027	0.256	-.039	0.099	-.096	<0.001
	**Diff**	***p***	**Diff**	***p***	**Diff**	***p***
Gender M	7.35	<0.001	0.019	<0.001	0.092	<0.001
DM	-23.6	<0.001	-0.366	<0.001	-0.054	<0.001
Smoker	-17.2	<0.001	-0.109	0.225	-0.001	0.951
CCS ≥ 2	-17.7	<0.001	-0.332	<0.001	-0.104	<0.001

Diff = mean difference between groups, r = Pearson correlation coefficient. Other abbreviations are the same as in Tables 1 and 2. In group comparisons, the values of SDNN, ln(LF) and DFA1 were higher in males, and lower in diabetes and in CCS class ≥ 2. The values of SDNN were lower in smokers.

### Association of heart rate variability measurements with different modes of death in patients with diabetes

In the subgroup analysis of 729 diabetic patients, a total of 161 (22.1%) patients suffered death. Of the deaths, 41 (5.6%) were caused by SCD/SCA and 39 (5.3%) by NSCD. Death attributable to other causes occurred in 81 (11.1%) patients. Several clinical variables differed significantly in univariate comparisons between the study groups, and after adjustments, in the multivariate Cox regression analysis (Table 4). When left ventricular end-diastolic diameter, left ventricular end-systolic diameter, LVEF and LVMI were tested in the Cox multivariate clinical model in a stepwise manner, LVEF and LVMI retained a significant association with SCD/SCA. When age, alcohol consumption, CCS class ≥2 and LVMI were entered into the Cox multivariate clinical model, age and CCS class ≥ 2 retained a significant association with NSCD. When age, gender, body mass index, alcohol consumption, right bundle branch block and left ventricular end-diastolic diameter were tested in the Cox multivariate clinical model, age, gender and right bundle branch block retained a significant association with NCD. HRV measurements were entered one at a time into the multivariate clinical model as a continuous variable (Table 5). SDNN retained significant association with risk of SCD/SCA and NSCD, ln(LF) and ln(VLF) with all modes of death, and DFA1 only with SCD/SCA. LF/HF ratio and PL slope lost their significant association with NSCD and NCD after relevant adjustments.

**Table 4:** Clinical characteristics and heart rate variability parameters in study patients with diabetes at baseline.

Variable	Alive (n = 568)	SCD/SCA (n = 41)	*p*	NSCD (n = 39)	*p*	NCD (n = 81)	*p*
Age	66.7 ± 8.3	68.9 ± 6.9	0.071	72.5 ± 8.6	<0.001	72.9 ± 7.3	<0.001
Gender M, (%)	68.2	75.6	0.309	64.1	0.598	81.5	0.007
BMI (kg/m^2^)	30.0 ± 4.8	29.9 ± 5.2	0.926	30.5 ± 6.1	0.474	28.7 ± 4.3	0.014
BP, systolic	147.9 ± 24.8	144.8 ± 23.5	0.419	144.9 ± 21.9	0.445	149.1 ± 29.1	0.685
BP, diastolic	80.8 ± 11.6	81.2 ± 11.1	0.810	78.3 ± 11.7	0.172	79.7 ± 13.7	0.466
Smoker (%)	8.8	17.1	0.111	2.6	0.387	4.9	0.118
Alcohol	1.9 ± 4.4	3.4 ± 8.6	0.375	0.6 ± 1.7	<0.001	1.1 ± 2.9	0.017
CCS ≥ 2 (%)	47.6	61.0	0.107	84.6	<0.001	55.6	0.157
LBBB (%)	2.7	4.9	0.298	2.7	1.000	2.5	1.000
RBBB (%)	5.0	4.9	1.000	5.4	0.709	11.4	0.012
β-blocker (%)	90.7	95.1	0.416	92.3	1.000	91.4	0.846
Ca-blocker (%)	32.8	31.7	1.000	43.6	0.161	35.8	0.616
ACEI/ARB (%)	77.1	75.6	1.000	76.9	1.000	75.3	0.780
Lipid l.m. (%)	91.5	87.8	0.383	82.1	0.040	85.2	0.037
Anti-t.m. (%)	97.5	97.6	1.000	92.3	0.066	100	0.0246
Anti-a.m. (%)	0.7	0.0	1.000	5.1	0.025	0.0	1.000
Diuretics (%)	46.0	48.8	0.749	61.5	0.049	61.7	0.003
Nitrates (%)	43.2	58.5	0.051	66.7	0.003	40.7	0.721
LVEDD (mm)	50.2 ± 6.6	53.6 ± 9.6	0.025	51.5 ± 8.7	0.328	48.8 ± 6.7	0.034
LVESD (mm)	32.8 ± 7.05	37.5 ± 10.7	0.005	34.9 ± 9.8	0.165	31.8 ± 7.3	0.187
LVEF (%)	63.7 ± 10.0	58.3 ± 13.9	0.013	61.2 ± 13.3	0.224	64.0 ± 10.8	0.739
LVMI (g/m^2^)	109.5 ± 27.9	125.2 ± 35.0	0.005	118.9 ± 30.3	0.030	113.5 ± 29.4	0.176
HR	68 ± 9	70 ± 11	0.134	66 ± 8	0.220	67 ± 10	0.576
SDNN (ms)	123.2 ± 40.5	106.6 ± 34.3	0.007	100.0 ± 32.7	<0.001	120.5 ± 45.6	0.520
ln(HF)	5.24 ± 1.03	5.04 ± 1.27	0.181	5.05 ± 1.09	0.222	5.26 ± 1.24	0.866
ln(LF)	5.69 ± 0.94	5.22 ± 1.26	0.017	5.20 ± 0.97	<0.001	5.46 ± 1.09	0.016
LF/HF ratio	2.01 ± 1.45	1.71 ± 1.46	0.173	1.48 ± 1.22	0.019	1.55 ± 1.16	<0.001
ln(VLF)	6.65 ± 0.76	6.33 ± 0.94	0.027	6.18 ± 0.75	<0.001	6.45 ± 0.84	0.012
DFA1	1.11 ± 0.24	1.01 ± 0.26	0.008	1.00 ± 0.25	0.003	1.03 ± 0.24	<0.001
PL slope	-1.37 ± 0.18	-1.40 ± 0.19	0.259	-1.45 ± 0.19	0.004	-1.40 ± 0.18	0.044
ApEn	0.91 ± 0.22	0.91 ± 0.22	0.968	0.90 ± 0.21	0.756	0.90 ± 0.24	0.445

Abbreviations are the same as in Tables 1 and 2. *p*-values when compared with all other study patients, i.e., with patients who remained alive/experienced other mode of death.

**Table 5:** Association of heart rate variability parameters with sudden cardiac death or sudden cardiac arrest, non-sudden cardiac death and non-cardiac death in the Cox regression analysis in the patients with diabetes.

		SCD/SCA			NSCD			NCD		
Variable		HR	95% CI	*p*	HR	95% CI	*p*	HR	95% CI	*p*
SDNN	uv	1.150	1.049–1.262	0.003	1.229	1.109–1.362	<0.001	1.032	0.974–1.094	0.282
	mv	1.117	1.023–1.219	0.014	1.188	1.070–1.318	0.001	1.040	0.982–1.102	0.180
ln(LF)	uv	1.791	1.310–2.447	<0.001	1.826	1.320–2.526	<0.001	1.408	1.119–1.771	0.004
	mv	1.551	1.128–2.133	0.007	1.545	1.119–2.132	0.008	1.247	1.003–1.551	0.047
LF/HF ratio	uv	1.219	0.938–1.583	0.138	1.513	1.092–2.097	0.013	1.394	1.129–1.721	0.002
	mv	1.165	0.912–1.489	0.220	1.119	0.821–1.526	0.478	1.111	0.903–1.366	0.320
ln(VLF)	uv	1.864	1.285–2.703	0.001	2.272	1.583–3.262	<0.001	1.559	1.182–2.055	0.002
	mv	1.501	1.041–2.166	0.030	1.940	1.296–2.905	0.001	1.439	1.084–1.910	0.012
DFA1	uv	1.201	1.065–1.355	0.003	1.236	1.092–1.399	<0.001	1.181	1.083–1.289	<0.001
	mv	1.141	1.009–1.290	0.035	1.105	0.959–1.273	0.167	1.072	0.973–1.182	0.160
PL slope	uv	1.118	0.941–1.327	0.204	1.290	1.083–1.535	0.004	1.143	1.013–1.291	0.031
	mv	1.053	0.892–1.242	0.542	1.153	0.961–1.383	0.126	1.071	0.939–1.221	0.309

Abbreviations are the same as in Tables 1 and 2. SDNN (every 10 decrease), LF/HF (every 1 decrease), ln(LF) (every 1 decrease), ln(VLF) (every 1 decrease), DFA1 (every 0.1 decrease), PL slope (every 0.1 decrease). *p*-values when compared with all other study patients, i.e., with patients who remained alive/experienced other mode of death. Significant clinical variables in the multivariate clinical model were LVEF and LVMI for SCD/SCA; age and CCS class ≥ 2 for NSCD; and age, gender and right bundle branch block for NCD.

## Discussion

The main finding of the present study was that several domains of HRV predicted an increased risk of SCD/SCA, NSCD and NCD in patients with CAD in the current treatment era. DFA1 and ln(LF) were associated with increased risk of SCD/SCA after adjustments with relevant clinical variables, but neither of the domains predicted a distinctively elevated risk of this mode of death. Generally, the HRV parameters failed to identify increased risk between a specific type of cardiac death and NCD, turning them into more overall predictors of death. Therefore, the clinical usefulness of HRV is limited in CAD patients in the current treatment era.

During the past decades, CHD mortality has decreased substantially almost all over the world, particularly in high-income countries [28]. Over these decades, breakthroughs have been made in evidence-based therapies, including new medicine and invasive treatment strategies, which have had a significant impact on decreasing mortality. It is estimated that approximately 45% of the decline in CHD mortality is attributable to medical and interventional/surgical treatments, of which secondary prevention and initial treatments for acute myocardial infarction or unstable angina have been among the most significant factors [19,20]. Nowadays, medications such as anti-thrombotic agents, lipid-lowering agents and beta-blockers are used extensively due to robust evidence of their favorable prognostic and anti-anginal influence [29,30]. Beta-blockers have an augmenting impact on HRV, which may be one of its action mechanisms and might decrease the predictive value of autonomic markers [31,32]. The vast majority of the patients in the present study were on beta-blocker medication. Percutaneous coronary intervention (PCI) and coronary artery bypass graft (CABG) are invasive treatment strategies which are routinely used in modern clinical settings and known to improve patients’ outcome and relive ischemic symptoms [29,30]. There is evidence that restored myocardial perfusion by PCI in CAD patients depresses HRV values instantly after operation, but HRV returns to the preoperative level and seems to trend beyond it [33,34]. After CABG, patients have permanently suppressed HRV levels but some domains of HRV return to preoperative values [35,36]. Therefore, it is suggested that HRV parameters may lose their predictive value after GABG due to HRV depressing and simultaneous survival improving effect. These influences are contradictory if HRV is used for risk stratification [37,38]. In the present study, a majority of the patients had prior PCI or CABG.

As discussed above, many of the modern evidence-based medical strategies have an effect on HRV parameters, whereas the risk of CHD mortality has been considerably reduced by using optimal strategies. Therefore, the association between HRV parameters and risk of SCD/SCA or NSCD in CAD patients might be relatively weaker in the current medical settings, which may partly explain why reduced HRV seems to be a more general indicator of mortality risk. Furthermore, it is plausible to speculate that patients with preponderant, particularly end stage, non-cardiac illness and increased cardiovascular risk profile represented by reduced HRV may die earlier [39].

Several previous studies have shown that reduced HRV predicts mortality in CAD patients with a prior myocardial infarction [5,10]. In a large study of post-myocardial infarction patients treated according to the modern guidelines, decreased HRV was found to predict both SCD and NSCD, an observation which is in line with our present findings in patients with stable CAD [40]. HRV measurements have also been observed to predict ventricular fibrillation or symptomatic sustained ventricular tachycardia in postinfarction patients [41]. Although there are some previous suggestions that nonlinear measurements of HRV, such as DFA1, could yield incremental prognostic information in postinfarction patients [12,13] and have a closer association with life-threatening ventricular tachyarrhythmias [15,42], none of the HRV measurements have been proven to be specific risk markers for SCD. In alignment with this concept, our present findings indicate that decreased HRV is rather a general risk marker for death than a specific indicator for the risk of SCD in the modern treatment era, and is therefore not helpful as a sole risk indicator for targeting ICD therapy to prevent SCD. Furthermore, patients with CAD need the best standard therapy including optimal medical therapy, and PCI or CABG, if needed, regardless of whether they have an increased risk profile or not as evaluated by HRV. Theoretically, it is possible that HRV could refine the risk for SCD if combined with information on other risk indicators including repolarization variability, such as the QT variability index [43]. In the present study, ln(LF) and DFA1 predicted SCD even after relevant adjustments; however, ln(LF) was also associated with risk of NSCD and NCD, and DFA1 with risk of NCD. Interestingly, concomitant activation of both vagal and sympathetic outflow typically decreases DFA1 [14], and some studies have suggested that an increase of sympathetic activity also can manifest as a decrease of the value of LF [44]. These autonomic nervous influences may increase the vulnerability to life-threatening ventricular tachyarrhythmias [45].

The association between reduced HRV and the risk for long-term mortality has been shown to be at least as strong in diabetic patients as in nondiabetic patients [46], an observation which is supported by our present findings. However, there has also been some controversy [38]. Decreased HRV values have been found in patients with diabetic neuropathy [47]. Concurring with these previous observations, the present patients with DM had lower values of HRV. It has earlier been shown that CAD patients with DM are at greater risk of SCD/SCA [21]. In the present analysis, decreased DFA1 values in patients with DM had closer specific association with the risk for SCD/SCA after relevant adjustments than the DFA1 values in all study patients.

Our study has some limitations. Our analysis was based on baseline HRV measurements. We did not perform control measurements of HRV during the follow-up. Therefore we were unable to evaluate, for example, aging-related changes in HRV, also considering that there were some differences in individual follow-up times.

Different domains of HRV are associated with increased risk of SCD, NSCD and NCD, but not distinctively with SCD in patients with CAD in the current treatment era. The clinical usefulness of HRV measurements alone in these patients is therefore limited.

## Supporting information

S1 File Minimal data set. (XLSX)
